# Mobile health apps: An exploration of user-generated reviews in Google Play Store on a physical activity application

**DOI:** 10.1177/20552076211014988

**Published:** 2021-05-08

**Authors:** Miznah Al-Abbadey, Megan M-W Fong, Laura J Wilde, Roger Ingham, Daniela Ghio

**Affiliations:** 1 Department of Psychology, University of Portsmouth, Portsmouth, UK; 2 Department of Psychology, University of Southampton, Southampton, UK; 3 Centre for Intelligent Healthcare, Faculty of Health and Life Sciences, Coventry University, Coventry, UK; 4 Primary Care, Population Sciences, and Medical Education, University of Southampton, Southampton, UK

**Keywords:** Mobile health, physical activity, apps, obesity, behaviour change techniques

## Abstract

**Objective:**

This study aimed to evaluate reviews that have been posted publicly on the app ‘MapMyRun’ to investigate which features were associated with usage of the app. A secondary aim was to determine whether MapMyRun consisted of specific behaviour change techniques that would have increased the likelihood of users being engaged with the app.

**Methods:**

Reviews posted on MapMyRun by users between 1st May 2017- 30th April 2018 were extracted, coded and analysed using content analysis.

**Results:**

Eleven behaviour change techniques were identified among the features of MapMyRun. A total of 3,253 reviews met the inclusion/exclusion criteria, and 12 codes were developed. The codes were grouped into 8 subthemes within 2 main themes: ‘Effort’ and ‘Self-monitoring’. Consistent with previous literature, ‘Goal-Setting’ and ‘Self-Monitoring of Behaviour’ were two techniques included in MapMyRun. Social features of MapMyRun facilitated competition among users, their family, and friends.

**Conclusions:**

This was the first qualitative review to assess a single mobile health physical activity app and analyse it from the perspectives of the users. Creators of future mobile health apps should focus on user friendliness and the use of social features, as both may increase the chances of users’ continued use with the app.

## Introduction

Obesity is a global public health issue and contributes to numerous health conditions including cardiovascular diseases, type 2 diabetes, musculoskeletal disorders, and cancers.^1^ Globally, obesity has nearly tripled since 1975 and approximately 13% of the world’s population were obese in 2016^2^. The cause of overweight and obesity ultimately comes down to an energy imbalance between calories expended and calories consumed.^2^ Therefore, a reduction in calorie dense foods and an increase in physical activity can assist in reducing and preventing overweight and obesity. However, this relies on individuals’ lifestyle choices and their ability to change habitual behaviour. Interventions that focus on dietary and physical activity changes are considered most effective for weight loss, although long term adherence remains poor.^3^

According to Abraham and Michie, there are common and distinctive behaviour change techniques (BCTs) across behaviour change interventions.^4^ The identification of distinct BCTs has improved the standardisation of behaviour change interventions, allowing interventions to be replicated. This has also led to improvements in identifying which BCTs, alone or in combination, are more effective. BCTs that have been found to be effective regarding weight outcomes include goal setting (behaviour), feedback on behaviour, self-monitoring of behaviour, and social support (unspecified).^5^

Mobile health, or mHealth, is a rapidly growing field with thousands of applications (apps) available to download with the focus on supporting and encouraging individuals to engage in positive lifestyle change.^6^ MHealth apps have been shown to be effective at reducing body weight and increasing physical activity compared with control interventions.^7^ MHealth apps may be particularly useful in promoting health because of inbuilt features including behavioural prompts, reminders, data recording, and social functionality.^8^ However, research is yet to keep up with the growing use of mHealth apps, and studies investigating their effectiveness is limited.^9^ Moreover, it is unclear how theoretically grounded current mHealth apps are. Research has shown that there is substantial variation in the number of BCTs present in popular mHealth apps with paid apps more likely to include techniques most commonly associated with greater effectiveness for behaviour change in relation to physical activity.^10^ Moreover, many mHealth apps are not necessarily designed by health professionals nor regulated.^7,11^ The accuracy and relevance of the information provided in the apps has been questioned.^12–14^ This is a concern as more health care professionals are recommending mHealth apps to promote healthy lifestyle behaviours, such as physical activity.^9,11^ MHealth apps that have been developed with health professionals tend to include higher quality information.^12^ Apps with lower health information quality have been found to provide either limited or overwhelming information and sometimes lack credible information.^12^

Middelweerd and colleagues evaluated the use of BCTs in ‘Health and Fitness’ apps that promote physical activity. A total of 62 apps were identified which included on average five BCTs (ranging from two to eight).^15^ These included self-monitoring, providing feedback on performance, and goal setting. The BCTs that were used in the apps aligned with the BCTs that were most commonly used in other types of physical activity promotion interventions.^15^ This study indicated that mHealth apps have the potential to effectively integrate tailored BCTs. However, there are still knowledge gaps regarding the effectiveness of mHealth apps, both for the users and for healthcare professionals. As health apps grow in popularity, it is important that they meet a certain level of scientific validity before being released to the general public.

The majority of studies looking at mHealth apps that aim to support physical activity have failed to examine the feedback or opinions from the users themselves,^15–17^ although a few studies have examined user reviews in the context of trying to understand what apps are available for weight loss and bipolar disorder.^18,^^19^ However, users’ experiences with mHealth apps aiming to support physical activity have not been explored in regard to what features users find most useful, and this may have implications for understanding what works to maintain app use to sustain behaviour. While certain BCTs have been found to be more effective at increasing physical activity and weight loss, research needs to consider their use in mHealth app alongside user experiences. A user-centred approach is important in determining how users interact with such apps and what features will maintain their engagement in the medium to longer term. By combining users’ perspectives and what is already known about specific BCTs, mHealth apps have the potential to effectively promote health on a large scale.

This study aimed to identify the BCTs used in MayMyRun (MMR) according to the taxonomy of BCTS.^4^ The study also aimed to identify – from the posted reviews - what features of the MMR app users report being helpful and unhelpful. The mHealth app, MMR by Under Armour, was selected for this study because it is popular (as evidenced by the number of downloads); it aims to increase users’ physical activity levels which is a widespread focus of behaviour change interventions.

## Methods

### MMR app

MMR is an mHealth app primarily used to track and map out running routes using GPS technology to help users reach their running goals. MMR is also able to create customisable training plans, personalised coaching tips, and provides real-time running updates including pace, distance, and elevation. In addition to running, users can log other physical activities such as cycling, walking, and gym workouts. MMR includes social features whereby users can see what their friends are doing via an ‘Activity Feed’. The social features allow users to share their workouts on social media platforms and join various competitive challenges. It has been installed by over 10 million users.

### Sample

Publicly available reviews and comments posted on the MMR app were extracted from the Google Play Store. The ‘Google Play Store’ was selected because the reviews could be accessed flexibly using a laptop or computer. This contrasts with the ‘App Store’, which restricts access to apps and their reviews to mobile devices. Ethical approval for use of secondary data was provided by the University of Southampton Ethics Review Committee (ERGO reference 41718).

There were 6,872 anonymous reviews and comments on MMR registered on the Google Play store between 1st May 2017 and 30th April 2018; these were all electronically extracted. Reviews posted on the Google Play store were selected because the Android operating system held a significant proportion of the global market at the time of the study (approx. 85% in 2018).^20^ Moreover, the format of the Google Play store meant that the reviews could be easily filtered and sorted. The date the reviews were posted and additional comments and replies from the app developers were also extracted. To be included, the reviews had to meet the following inclusion criteria: be written in English; published between 1st May 2017 and 30th April 2018; consist of at least one sentence with a minimum of five words; discusses a feature of MMR; include some critical evaluation of the app’s features (i.e., not only describe the MMR as being ‘good’ and/or ‘bad’); and include an overall opinion of MMR, referring to what the user liked or disliked.

### Data analysis procedure

**Figure 1.**
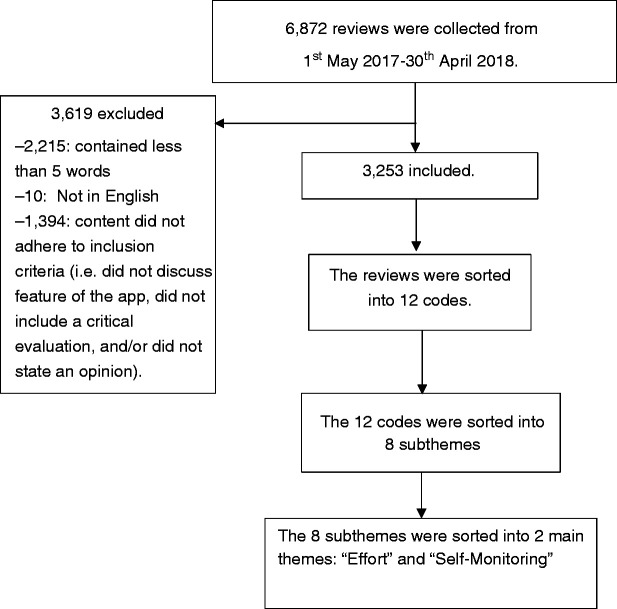
Flow chart of reviews to themes.

Once all the information from the reviews was imported into an Excel document, content analysis was conducted using the methodology as described by Hsieh & Shannon.^21^ Content analysis is a study framework whose purpose is to describe a phenomenon and was used to identify patterns in the data.^21,22^ An inductive approach was used; i.e., analysis was data-driven rather than based on any pre-existing coding frame. Posted reviews were initially read carefully to identify meaningful sections of texts, which were allocated codes that reflected the underlying meaning of the users’ comments on their use of MMR. Each included review was given at least one code, with some having multiple codes. Codes were then grouped together into themes.^23^ Reviews that did not meet the inclusion criteria were simply coded as ‘excluded’ (See Figure 1). A codebook was developed to ensure data were coded consistently and was iteratively designed throughout the analysis process to reflect the data.^24^ Regular team meetings (MF, MAA, RI, DG) were arranged to test and discuss the codebook before coding was completed to ensure transparency. The final codebook used in the analysis is shown in Table 1. The codebook includes content variables that relate to a variety of features included in the MMR app. The codes defined in Table 1 were used to code all the extracted reviews. Descriptive statistics were used to summarise the distribution of the data across the codes. The mean and standard deviation (SD) were used to present the average number of reviews per code. Additional demographic information of the participants could not be obtained because the reviews were anonymous.

**Table 1.** Final codebook used to categorise data.

Codes	Definition	Examples
Tracking	Comments relating to how well the application tracks their run. E.g., using real time mapping, distance, time, and calories burned	“I love this app. It lets me keep track of my pace and distance easily”
Navigation	Comments on the usefulness of the listed routes	“Great outdoor tracker. Helps you find good runs/walks/hikes wherever you are.”
Personalise	Commenting on how easily users can customise their own workout	“Maintains a steady signal and a plethora of adjustable options with which to create a personalized trainer/workout routine from.”
Syncs	Comments concerning how well the app can synchronise with other apps or devices	“Great app and it syncs all of my walks runs and workouts to my fitness pal for easier calculations”
Interface	Comments relating to the app’s interface and usability	“Great app. Simple to use.”
Audio Feedback	Comments relating to the audio notifications during workouts, including split time, distance, and overall pace	“Thanks for updates every mile”, “Great app, let's me know my mile pace.”
Logging	Comments concerning the app’s feature that allow users to log data	“Great way to log workouts”, “Great for tracking runs. Has option to add notes after workout.”
Accuracy	Comments relating to the accuracy of the app	“Accurately gives distance and pace.”
Planning	Comment about the apps feature that allow users to plan their workouts or set goals	“Only thing missing is that I can't create or plan out routes beforehand like I can on the website”
Suggestions for Improvement	Comments that suggest changes for improvement	“Bring back the elevation stats.”
Social	Comments relating to the social features on the app	“I like the comments section.”

Users who posted a review could also include an associated star rating that indicated their overall outlook on the app. The star ratings ranged from one to five stars with more stars indicating a more positive rating. The star ratings and the content of the posts were used to categorise each review as either ‘positive’ or ‘negative’ on a five-point scale. Ratings for positive or negative reviews were discussed and reviewed by each member of the team (MAA, MF, RI, DG) for the first 10% of the reviews to establish review categories. Agreement on how to categorise the reviews allowed MF to continue categorising the rest of the dataset. Reviews were considered positive if the majority of the content was optimistic and the star rating was 3 or higher out of 5, while negative reviews were predominantly pessimistic and had a star rating of 1 or 2.

Two researchers (MF, LW) downloaded MMR independently and applied the BCT taxonomy (V.1^4^) to identify specific BCTs included as inbuilt features of MMR as of version 20.2.0. At the time of this study, MF and LW were active users of MMR and very familiar with the app’s features. MF and LW independently coded for BCTs present in the app using the BCT taxonomy. The number of times the specific BCT appeared within the app was not recorded; if the BCT was present in one instance this was enough for the BCT to be coded as present. BCTs were coded as present according to definitions outlined by Michie et al.^4^ Discrepancies were discussed with the team and decisions were made based on what the app offers (e.g. did not include the BCT “Biofeedback” because this requires syncing with other devices). MF and LW’s knowledge of the app fed into the research team’s discussions of the coded BCTs and enhanced the team’s independent evaluation of MMR. The BCTs were also used to examine the main behaviour change ‘clusters’ within the BCT taxonomy from the perspectives of the users.

## Results

A total of 3,253 reviews met the inclusion criteria and were included for the main analyses (see Figure 1).

Descriptive statistics summarising the data across all 12 content categories is shown in Table 2. Among the 12 codes, ‘Tracking’ was the most common with 1,422 reviews, while ‘Personalise’ was the least common. The mean number of reviews per code was 368.50 (SD = 369.00).

**Table 2.** Content analysis of codes.

Codes	Total # of reviews	# of Positive* reviews	# of Negative** reviews
Accuracy	360	277 (76.9%)	83 (23.1%)
Audio Feedback	332	280 (84.3%)	52 (15.7%)
Interface	629	595 (94.6%)	34 (5.4%)
Logging	324	295 (91.0%)	29 (9.0%)
Navigation	188	145 (77.1%)	43 (22.9%)
Personalise	10	10 (100%)	0 (0%)
Planning	54	43 (79.6%)	11 (20.4%)
Social	154	125 (81.2%)	29 (18.8%)
Suggestions for Improvement	298	154 (51.7%)	144 (48.3%)
Syncs	294	212 (72.1%)	82 (27.9%)
Tracking	1422	1025 (72.1%)	397 (27.9%)
*Total Reviews*	4,065 (1,168 overlap with one or more codes)	3,161 (78%)	904 (22%)


*Positive: Star rating of review is 3 or greater and is mainly optimistic.

**Negative: Star rating of review is 3 or less and is mainly pessimistic.

### Behaviour change techniques

Table 3 provides a summary of the standardised BCTs identified in MMR through independent exploration by the research team. A total of 11 individual BCTs were identified with the largest proportion being from the ‘Goals and planning’ cluster and ‘Reward and threat’ cluster. A large proportion of the BCTs included were thematically social despite spanning across different behaviour change clusters. This included ‘3.1 Social support’, ‘6.2 Social comparison’, and ‘6.3 Information about others’ approval’. This is reflected in the user reviews, as a high proportion (81.2%) of reviews that referred to social elements of MMR were positive (Table 2).

As discussed, the largest proportion of the reviews were coded under ‘Tracking’, which fall into the ‘2.2 Feedback on behaviour’ and ‘2.3 Self-monitoring of behaviour’ BCTs. The theme ‘Self-monitoring’ further supports the importance of the ability to self-monitor progress for users. Overall, all the identified BCTs have been referred to in the reviews, suggesting they each had some impact on the users’ experience with MMR. This is indicated by the column ‘Supporting user review’.

### Key themes

Based on the reviews and star ratings, there were more positive than negative reviews overall; out of all the reviews, 78% were positive. Users spoke highly of MMR’s user interface, as 94.6% of the reviews that mentioned the interface were positive. The ‘Tracking’ feature was reviewed the most with only 27.9% of them being negative. The content analysis resulted in two main themes: ‘Effort’ and ‘Self-Monitoring’. Both themes comprised four subthemes, which are summarised in Table 4.

**Table 3.** Behaviour change techniques in MMR as identified by the research team.

Cluster	BCT	Description	Supporting user review
(1) Goals and planning	1.1 Goal setting (behaviour)	Can set goals related to number, distance or duration of activity, such as run, ride or walk for ‘this week’ or ‘next week’	*Tracks my distance, route, elevation and speed. I can also set goals and share with friends (Review 3975)*
	1.4 Action planning	You can set a plan, but this is an additional purchasable feature of the app. You can set specific dates to do your activity, e.g. long runs, short runs etc on certain days of the week.	*Also great for planning out routes beforehand if you want to make sure you run a certain distance (Review 1496)*
(2) Feedback and monitoring	2.2 Feedback on behaviour	Provides feedback on distance, calories, time, pace, path etc.	*I use this for mapping my mileage, splits, etc. It connects to phone GPs for real-time feedback (Review 4248)*
	2.3 Self-monitoring of behaviour	Self-monitors workouts/runs etc.	*I use this app only for tracking runs/walks/hikes, which it does very well (Review 5996)*
(3) Social support	3.1 Social support (unspecified)	Social support is available from friends or family also using the app and you can share workouts to other social media platforms on the app	*I like being linked with friends and family members who also run or walk using this app. It's a nice tool (Review 6053)*
(6) Comparison of behaviour	6.2 Social comparison	Users can see other user’s activity data that they are friends with and not friends with	*Excellent way to track your workouts and compete against yourself and friends. Friendly competition is very effective in helping me stay motivated to exercise (Review 2023)*
	6.3 Information about others' approval	Other users can comment or like their workouts	*I love it, I can keep track of all my runs and my friends, my friends comments keep me motivated thanks mmr (Review 6790)*
(7) Associations	7.1 Prompts/cues	Can add prompts/reminders for specific times of the day to remind you to do your workout/goals	*Easy to use. Like the voice prompts (Review 2088)*
(10) Reward and threat	10.1 Material incentive (behavior)	For US participants only, they have a chance to win certain UnderArmour items if they complete certain goals.	*Love it!! Tracks well, link to UnderArmour got me some great new shoes, discounts for distance; You can't beat that with a stick!!! (Review 2994)*
	10.2 Material reward (behavior)	For US participants only, they have a chance to win certain UnderArmour items if they complete certain goals.	
	10.3 Non-specific reward	Achievements and badges can be gained by joining challenges and reaching goals or paying for additional features. Other users can comment or like.	*Great rewards and incentives to keep you looking forward (Review 3429)*

Note: “BCT” = Behaviour change technique; (*N*) = Behaviour change cluster number

**Table 4.** Main themes and subthemes.

Themes	Subthemes	Example review
Effort	Functionality	“This app tracks my run each day. It's reliable and useful. I like the pause feature as I run with my dog.”
	Compatibility	“I use it with my Samsung watch. Works perfectly and provides accurate feedback/tracking.”
	Customisability	“Great personalized running plans - great app!”
	Route Options	“Quick and easy to map out a run, especially when traveling to someplace unfamiliar.”
Self-monitoring	Feedback	“It is accurate, and it notifies you after every mile which I like because I don't have to check. It also gives you clear stats on your run. I love it!”
	Diary	“GOOD diary to record my self (sic) … knew to this town plus just started running …”
	Social	“I like being linked with friends and family members who also run or walk using this app. It's a nice tool.”
	Goal-Oriented	“Great for monitoring progress towards goals in a variety of activities. Helps monitor pace and helps reach beyond what you think you are capable of.”

#### Effort

Many reviews commented on the accessibility of MMR and how user-friendly the various functions are. The easier it was to use MMR, the less ‘effort’ required by users to learn how to use the app (e.g., *“This app is awesome. It helps me to know where I'm at with my fitness goals without me having to do all the calculating*.” [Review 5488]). Approximately a third of the reviews referred to the functionality of the app, which included comments on the user interface, accuracy of the GPS feature and the reliability of the reported statistics:

*“Have used this app off/on for a few years now and I really like the new features. Finding that its more accurate on miles tracking than my gps watch!!!” (Review 4276)*

*“I have been doing road and trail running and only this app accurately maps my run. The others [other apps] do not get GPS coordinates quick enough to be accurate, being up to 500 m under the true distance. This app also gives accurate current pace based on a very local distance rather than over the last km.” (Review 3409)*

Examples of the reported statistics within the app included distance run by the user and the number of calories burned. Overall, comments suggested the users felt the reported statistics were accurate and positively regarded.

Reviews commented on MMR’s interface and how easy it was to navigate. Most of the reviews suggested MMR’s interface was intuitive with only a minority of reviews suggesting it was too complicated. Many comments referred to the compatibility of MMR with other apps or devices. Overall, the reviews suggested users preferred MMR to be compatible with other apps as well as their phones (for example: *“Works well, good to partner it with a calorie tracker. My Fitness Pal has been good for me” [Review 1475]).* In addition, about 15% of reviews suggested different ways users would prefer MMR to be more customisable. Users often commented on whether the app was able to provide what they wanted by tailoring aspects of the app. Recurring requests included having more personalised training plans and more customisability over the interval workouts option.

Mapping a user’s run or workout is a key part of the MMR app. MMR allows users to pick different ways to track the route they want to use during their workout, giving users the option to make mapping their run easier. Many reviews suggested users enjoyed using the pre-made routes, while others enjoyed using previous routes they ran which were recorded on the app. However, some reviews criticised the app for not allowing users to pre-plan a route before a run:

*“Good app but you can't create and plan courses with app (although you can do it online)” (Review 1551) *

#### Self-monitoring

Another key theme related to how well users were able to monitor their progress using the app. Users’ feedback on both the audio and statistics provided at the end of workouts was positive, for example:

*“It is accurate, and it notifies you after every mile which I like because I don't have to check. It also gives you clear stats on your run. I love it!” (Review 260)*

The audio feedback is provided during the workout and updates users on how long they have been running, the distance ran, their current pace, and average pace. At the end of the run, users are provided with written statistics including distance, duration, average pace, calories burned, elevation gain, and split times. Of the reviews, 84% of users that commented on audio feedback indicated they found it helpful, while the few negative reviews related to issues with the ‘voice’ used to deliver the feedback. This mainly consisted of technical issues such as not receiving audio feedback despite being enabled or there being a delay in the feedback.

After tracking their workout, users receive additional statistical feedback. Comments suggest this is useful as it provides them with an overall summary of their run and allows them to assess where they could have done better:

*Great app. Does everything I need so I can keep track of my workouts, and maintains a weekly summary for my records. (Review 2495)*

Moreover, users have the option to keep a diary of their workouts by saving them on MMR. Once saved, users may review their workouts and evaluate their improvements. MMR also allows users to compare their progress with average users matched by age and gender overall. This includes the ability to track their own and friends’ progress. The reviews suggest that users viewed the diary, the ability to compare progress with average users, and being able to track friends’ progress positively.

MMR includes a social feature that allows users to connect with family, friends, as well as other users on the app. Many reviews commented on how the social feature helped them stay motivated, for example:

*“Great tool! Pushes me to be fit, and my friends connected on the app push me too!” (Review 5256)*

Reviews also suggested that users enjoyed receiving praise from others, which reinforced their fitness efforts. Users can support and praise each other’s’ successes by posting workouts on a public newsfeed for other users to comment on, which suggests praise can be received from users who are not necessarily from individual’s contact list. Users are also able to compete in challenges with friends and family, or other users within their location. The challenges can be created by users or are sponsored challenges that are generated by MMR. The reviews suggest that users particularly valued the social features of MMR with over 81% of the comments that referred to social elements being positive:

*“Social aspect is great! My friends and I set up a workout challenge every month and it's the only thing that motivates me (Review 2023)*

Finally, many reviews discuss how MMR helped users create and achieve physical activity goals. The ‘goal’ feature on MMR updates users on their progress and when they have achieved their goals. Reviews indicated that users valued this feature as it helped them stay motivated and on track in line with their personal goals:

*“Great for monitoring progress towards goals in a variety of activities. Helps monitor pace and helps reach beyond what you think you are capable of.” (Review 2848)*

## Discussion

### Summary of findings

The study aimed to identify whether inbuilt features of MMR consisted of specific BCTs and to explore what features users report being most helpful and unhelpful using user-generated comments. A total of 11 BCTs were identified with a large proportion consisting of social attributes, goal setting, self-monitoring and feedback. The BCT identified fall within the following behaviour change clusters: ‘Goals and planning’, ‘Feedback and monitoring’, ‘Social support’, ‘Comparison of behaviour’, ‘Associations’, and ‘Reward and threat’.^4^ The current study found that, overall, the user reviews commented on all the identified BCTs which suggests they had a considerable effect on the users’ experience with MMR.

The user-generated reviews were also analysed using content analysis to assess MMR from the point of the users themselves. A total of 3,253 reviews were included and provided further insight into how users benefitted from the BCTs and the app in general. Overall, users commented on MMR’s functionality, compatibility with other applications, ability to create running routes, and the feedback functions. Although the reviews focused on different aspects of MMR, a large proportion referred to the ‘ease of use’ or the ‘effort’ required to use the app’s features. In addition, a significant proportion of the comments focused on the ‘self-monitoring’ features of MMR, including the app’s ability to provide feedback of progress, the diary, and goal-setting features. This also included MMR’s social features, which enabled users to monitor their progress through social comparison and social support.

### Study findings and wider literature

MHealth apps have been shown to be effective at promoting weight loss and increasing physical activity, which is most likely due to inbuilt features including behavioural prompts and their wide reach.^7,8^ The current study supports previous research suggesting that long-term use of mHealth apps requires simplicity, efficiency and enjoyment.^25^ Prioritisation of low user burden was evident in the reviews of MMR, and is also supported by previous qualitative work exploring user views about health-related smartphone apps.^26^ MMR features that were reviewed most positively (based on the frequency of positive reviews) include goal planning and social support. These features appear to be highly regarded as they are also present in other high quality apps (as rated using the Mobile Apps Rating Scale).^27^

Based on the findings, it is important for mHealth apps to minimise user burden as much as possible by reducing cognitive or mental effort. Cognitively demanding tasks can negatively impact task performance and mediates the behavioural consequences of motivation.^28^ According to Garbarino and Edell,^29^ cognitive effort is considered to be costly and people prefer to use minimal effort to make satisfactory decisions. Effort theories also suggest people search based on where they would expect the content to be, but the longer they have to search, the more ‘effort’ is involved.^30^ This is supported by findings from the current study as comments suggested users tend to only use the basic features of MMR as this is found more easily. Research has also shown that difficulties navigating a website can greatly reduce users’ satisfaction due to greater mental effort.^31^ As was stated by Stoll and colleagues, the more mental effort one puts into using an app, the less likely they will like it or continue to use it.^32^ Similarly, reviews from the current study that suggested MMR was difficult to use tended to have a lower associated star rating.

Many of the user-generated comments on MMR suggested the social features on the app increased their motivation to engage in physical activity. While research has suggested social support can be important in supporting weight loss, fewer than 20% of apps available in the market include social support.^18^ Findings from the current study suggest the inclusion of a community feature in an app that aims to monitor physical activity can increase sustainability in use of app and behaviour. Social features may have been perceived positively by increasing motivation through competition and by increasing social support, and social modelling.^33,34^ Receiving positive support from family and friends has been shown to support individuals initiate and maintain physical activity over time.^35^ Other research has shown that participants enjoy sharing information about their workout with others on social media as this provides them with social support and approval.^36^ These findings are consistent with how MMR users may connect with friends and family to give each other support.

### Implications

mHealth has rapidly increased in popularity and is the fastest developing eHealth sector.^37,38^ Yet, very few have been developed by professional sources and the majority have not been scientifically supported for their effectiveness, nor have they been validated for their health outcomes.^38,39^ Thousands of physical activity focused apps have been developed and their wide reach provides users the potential to self-manage their health at low cost.^38^ Incorporating specific BCTs can help encourage users self-manage their health.^40^ Given the high rates of obesity worldwide, there is a need for effective anti-obesity interventions that are easily accessible. mHealth has the potential to address this need but more evidence is needed to support their use.

Individuals usually use mHealth apps to either become or to stay healthy.^41^ The results from this study suggest that incorporating BCTs may help keep users engaged, which is a precondition to its effectiveness.^42^ Engagement with mHealth apps has been defined as “the extent (e.g. amount, frequency, duration, depth) of usage and the subjective experience characterised by attention, interest and affect”.^43^ While user-engagement was not specifically investigated, the user-generated reviews suggest various features of MMR, including the identified BCTs, may have enhanced user engagement with the app. These include features such as goal setting, feedback, and self-monitoring, social support, and rewards and incentives.^43^ To become an effective mHealth app, it is vital that users have a high level of engagement with MMR. Ensuring the interface is simple to navigate is important to keep people engaged with the app. More research is needed to investigate the relationship between cognitive effort and engagement with smartphone apps.

In this study, we utilised a novel and practical method to explore users’ experiences of an mHealth app by using user-generated reviews that are publically available. This method allowed us to easily collect a vast amount of data in a relatively short time frame without needing to spend time on research-related admin such as recruitment, transcribing, scheduling interview slots etc. In addition, because the reviews were posted anonymously, they are unlikely to reflect social desirability and app users could reflect and share their thoughts on the app freely. In contrast to traditional qualitative interviews, this method also provides researchers with the flexibility to focus on either longer or shorter time spans. Given the rapid growth of mHealth and frequent updates of individual apps, the use of user-generated reviews may be a feasible way to assess changing features over time.^38,44^ While collecting data through the use of user-generated reviews has its advantages, this method does not provide researchers the opportunity to probe participants and expand on the points they have made. The majority of the reviews are relatively short and lack the depth that would otherwise be gained through qualitative interviews. Further research could utilise more in-depth qualitative research techniques to explore the implications of the use of BCTs in mHealth and the potential facilitators and barriers of their use.

Although our findings relate to specific features of MMR, the results can be applied to other apps that are designed to increase physical activity using self-monitoring features. A large proportion of the reviews focused on ‘usability’ features of the app or ‘ease of use’, which can be applied to mHealth more widely. Depending on the number of user-generated reviews available, this method can also be used to evaluate users’ experiences of less popular or academia-developed apps either as part of a mixed-methods design or as the primary research method. Academia-developed apps face several major challenges at both the development and maintenance stages in app production. They are typically funded by research grants, which consist of high monetary cost, time, and resources needed to develop the app and there is usually a lack of funds available for graphic design and long-term maintenance.^44^ Moreover, large samples sizes are needed for research studies that evaluate academia-developed apps. While the current methodology does not provide the answer to all of these challenges, it may serve as a useful cost-effective and quick tool to support the evaluation of mHealth apps that have been developed.

### Strengths and limitations

This study had a number of limitations. The reviews gathered were solely from the Google Play Store, which meant users from other app stores such as Apple, Amazon, Windows, and Blackberry were not investigated. Including reviews from these additional app stores was beyond the scope of this study. However, future studies may consider including data from multiple app stores to ensure various viewpoints are represented. Only reviews written in English were included in the study and so the findings cannot be generalised to non-English speaking users of MMR. Moreover, because data consisted of reviews left on the Google Play Store, demographic data could not be obtained. Key demographic information, such as age and gender, would have provided useful insights into the users of the app. However, accessing data with this approach allowed for an exceptionally large dataset of users of the app. While collecting user reviews did not provide the depth and opportunity to further prompt users in ways that interviews might, the exploration by researchers and the size of the data set nevertheless helped to provide a comprehensive review of MMR. This is the first study to look at user reviews of a physical activity tracking app and gave insight into needs of the users and what they are looking for in a physical activity app.

## Conclusions

This study involved the in-depth analysis of a single physical activity app from the users’ point of view. This is the first qualitative study to examine users’ opinions of a mHealth app and investigated which app features users find to be most helpful.

The analysis resulted in two themes. The effort theme suggests users may be more likely to engage with mHealth if less cognitive effort is required, however more research is needed. This means ensuring the app is intuitive to use and user-friendly. Based on our findings, the use of self-monitoring techniques may enhance user engagement and the effectiveness of mHealth. More experimental research approaches are needed to investigate specific causal links. The self-monitoring theme suggests users appreciate the ability to track their own progress. Moreover, social features of MMR that support users with a community and encouragement may help keep users motivated to use the app.
